# Supplementary material for: Plasma Malondialdehyde and Risk of New-Onset Diabetes after Transplantation in Renal Transplant Recipients: A Prospective Cohort Study
Source: J Clin Med. 2019 Apr 4;8(4):453. doi: 10.3390/jcm8040453 (PMC6518172; doi:10.3390/jcm8040453)
Supplement: Supplementary file 1 [file jcm-08-00453-s001.pdf]

Supplemental Table S1. Plasma MDA concentration and all-cause mortality in RTR (n = 516).

All-cause mortality	HR (95% CI) per SD	*p*
Crude model	0.96 (0.73–1.25)	0.74
Model 1	0.97 (0.72–1.29)	0.81
Model 2	0.94 (0.70–1.26)	0.67
Model 3	0.93 (0.69–1.25)	0.62
Model 4	0.98 (0.72–1.32)	0.88
Model 5	0.96 (0.71–1.30)	0.80
Model 6	0.97 (0.71–1.30)	0.81

In total 86 (17%) RTR died. Model 1: crude model plus adjustment for demographic and anthropometric characteristics. Model 2: model 1 plus adjustment for metabolism-related variables. Model 3: model 2 plus adjustment for lifestyle characteristics. Model 4: model 3 plus adjustment for transplantation-related data. Model 5: model 4 plus adjustment for immunosuppressive therapy. Model 6: model 5 plus adjustment for inflammation.

Supplemental Table S2. Plasma MDA concentration and cardiovascular mortality in RTR (n = 516).

Cardiovascular mortality	HR (95% CI) per SD	*p*
Crude model	0.69 (0.38–1.24)	0.21
Model 1	0.70 (0.38 –1.31)	0.27
Model 2	0.64 (0.33–1.24)	0.18
Model 3	0.63 (0.32–1.24)	0.18
Model 4	0.65 (0.33–1.31)	0.23
Model 5	0.63 (0.31–1.27)	0.19
Model 6	0.62 (0.30–1.28)	0.20

In total 29 (6%) RTR died from a cardiovascular cause. Model 1: crude model plus adjustment for demographic and anthropometric characteristics. Model 2: model 1 plus adjustment for metabolism-related variables. Model 3: model 2 plus adjustment for lifestyle characteristics. Model 4: model 3 plus adjustment for transplantation-related data. Model 5: model 4 plus adjustment for immunosuppressive therapy. Model 6: model 5 plus adjustment for inflammation.

Supplemental Table S3. Plasma MDA concentration and dead-censored graft failure in RTR (n = 516).

Graft failure	HR (95% CI) per SD	*p*
Crude model	0.89 (0.65–1.23)	0.49
Model 1	0.90 (0.66–1.23)	0.50
Model 2	0.86 (0.62–1.19)	0.36
Model 3	0.83 (0.60–1.16)	0.28
Model 4	0.93 (0.68–1.28)	0.66
Model 5	0.94 (0.69–1.29)	0.71
Model 6	0.94 (0.69–1.29)	0.71

In total 57 (11%) RTR developed graft failure. Model 1: crude model plus adjustment for demographic and anthropometric characteristics. Model 2: model 1 plus adjustment for metabolism-related variables. Model 3: model 2 plus adjustment for lifestyle characteristics. Model 4: model 3 plus adjustment for transplantation-related data. Model 5: model 4 plus adjustment for immunosuppressive therapy. Model 6: model 5 plus adjustment for inflammation.
